# Psychosomatic Rehabilitation Patients and the General Population During COVID-19: Online Cross-sectional and Longitudinal Study of Digital Trainings and Rehabilitation Effects

**DOI:** 10.2196/30610

**Published:** 2021-08-26

**Authors:** Franziska Maria Keller, Alina Dahmen, Christina Derksen, Lukas Kötting, Sonia Lippke

**Affiliations:** 1 Department of Psychology & Methods, Jacobs University Bremen gGmbH Bremen Germany; 2 Dr. Becker Klinikgruppe Cologne Germany

**Keywords:** mental health, COVID-19, medical rehabilitation, psychosomatic rehabilitation, internet-delivered digital trainings

## Abstract

**Background:**

The COVID-19 pandemic has largely affected people’s mental health and psychological well-being. Specifically, individuals with a pre-existing mental health disorder seem more impaired by lockdown measures posing as major stress factors. Medical rehabilitation treatment can help people cope with these stressors. The internet and digital apps provide a platform to contribute to regular treatment and to conduct research on this topic.

**Objective:**

Making use of internet-based assessments, this study investigated individuals from the general population and patients from medical, psychosomatic rehabilitation clinics. Levels of depression, anxiety, loneliness, and perceived stress during the COVID-19 pandemic, common COVID-19–related worries, and the intention to use digital apps were compared. Furthermore, we investigated whether participating in internet-delivered digital trainings prior to and during patients’ rehabilitation stay, as well as the perceived usefulness of digital trainings, were associated with improved mental health after rehabilitation.

**Methods:**

A large-scale, online, cross-sectional study was conducted among a study sample taken from the general population (N=1812) in Germany from May 2020 to April 2021. Further, a longitudinal study was conducted making use of the internet among a second study sample of psychosomatic rehabilitation patients at two measurement time points—before (N=1719) and after (n=738) rehabilitation—between July 2020 and April 2021. Validated questionnaires and adapted items were used to assess mental health and COVID-19–related worries. Digital trainings were evaluated. Propensity score matching, multivariate analyses of covariance, an exploratory factor analysis, and hierarchical regression analyses were performed.

**Results:**

Patients from the psychosomatic rehabilitation clinics reported increased symptoms with regard to depression, anxiety, loneliness, and stress (*F*_4,2028_=183.74, *P*<.001, *η^2^_p_*=0.27) compared to the general population. Patients perceived greater satisfaction in communication with health care professionals (*F*_1,837_=31.67, *P*<.001, *η^2^_p_*=0.04), had lower financial worries (*F*_1,837_=38.96, *P*<.001, *η^2^_p_*=0.04), but had higher household-related worries (*F*_1,837_=5.34, *P*=.02, *η^2^_p_*=0.01) compared to the general population. Symptoms of depression, anxiety, loneliness, and perceived stress were lower postrehabilitation (*F*_1,712_=23.21, *P*<.001, *η^2^_p_*=0.04) than prior to rehabilitation. Psychosomatic patients reported a higher intention to use common apps and digital trainings (*F*_3,2021_=51.41, *P*<.001, *η^2^_p_*=0.07) than the general population. With regard to digital trainings offered prior to and during the rehabilitation stay, the perceived usefulness of digital trainings on rehabilitation goals was associated with decreased symptoms of depression (*β*=–.14, *P*<.001), anxiety (*β*=–.12, *P*<.001), loneliness (*β*=–.18, *P*<.001), and stress postrehabilitation (*β*=–.19, *P*<.001). Participation in digital group therapy for depression was associated with an overall change in depression (*F*_1,725_=4.82, *P*=.03, *η^2^_p_*=0.01) and anxiety (*F*_1,725_=6.22, *P*=.01, *η^2^_p_*=0.01) from pre- to postrehabilitation.

**Conclusions:**

This study validated the increased mental health constraints of psychosomatic rehabilitation patients in comparison to the general population and the effects of rehabilitation treatment. Digital rehabilitation components are promising tools that could prepare patients for their rehabilitation stay, could integrate well with face-to-face therapy during rehabilitation treatment, and could support aftercare.

**Trial Registration:**

ClinicalTrials.gov NCT04453475; https://clinicaltrials.gov/ct2/show/NCT04453475 and ClinicalTrials.gov NCT03855735; https://clinicaltrials.gov/ct2/show/NCT03855735

## Introduction

### Mental Health and the COVID-19 Pandemic

The COVID-19 pandemic has led to rapid changes in the lives of people all over the world, thus affecting both physical health as well as mental health and well-being [1]. Worries about one's own health, the health of family and friends, as well as worries associated with the future are indicative of decreased mental health and psychological well-being. Hence, for many individuals, the COVID-19 pandemic evoked feelings of uncertainty, social isolation due to contact regulations, stress reactions, symptoms of depression and anxiety, and general fear of the virus [2,3]. In case of prolonged concerns or worries, individuals are at risk of developing serious mental health disorders [4].

A study by Wang et al asked respondents to assess the psychological impact of the COVID-19 pandemic on their mental health. Results highlighted that 54% of the respondents rated the psychological impact of the COVID-19 pandemic as moderate to severe. Further, 29% estimated their own anxiety symptoms to be between moderate and severe, and 17% estimated symptoms of depression as moderate to severe [5]. Another study by Sønderskov et al revealed lower psychological well-being in the general public compared to before the COVID-19 pandemic [6]. Recent studies from the United States highlighted the worldwide increase in depressive symptoms as well as in symptoms of anxiety, which occurred about three times more frequently during the COVID-19 pandemic than before. Research has indicated that pre-existing mental health conditions may worsen due to COVID-19 [7,8].

The conjectured decrease in mental health worldwide may be explained by two developments associated with the ongoing course of the COVID-19 pandemic. On the one hand, the ramifications associated with the COVID-19 pandemic, such as uncertainties, unemployment, short-term employment, or social isolation, may pose a mental health threat. A cross-national comparison of Norway, the United Kingdom, the United States, and Australia found that secure employment status was associated with lower levels of loneliness and mental health distress as well as higher levels of well-being and quality of life during the early social distancing requirements of the pandemic [9]. Correspondingly, returning to work during the pandemic was associated with low levels of psychiatric problems [10].

On the other hand, the way most people live, work, study, socialize, or travel has been abruptly disrupted or shifted online. The associated containment measures, such as quarantining and physical distancing, restrict people in their freedom, but are necessary to control the disease's spread. Literature has shown that quarantining or physically distancing oneself from others may lead to problems associated with a decreased mental health status [11]. It can precipitate feelings related to fear, anger, anxiety, or even panic about possible negative outcomes and is associated with increased perceived loneliness and boredom.

The World Health Organization (WHO) has also expressed concerns with regard to the mental health and psychological well-being of individuals due to containment measures. According to the WHO, restrictions may interfere with people's daily activities and routines and may consequently lead to an increased perception of loneliness, depression and anxiety, insomnia, substance misuse, self-harm, or even suicidal behavior [12]. It has been shown that increased loneliness and reduced interactions due to social distancing are risk factors for several mental health disorders, such as depression, anxiety, and schizophrenia. Especially for women, young people, and those living with young children, mental health problems have increased over time [13].

### COVID-19 and Patients With Pre-existing Mental Health Disorders

Literature on the impact of the 2003 severe acute respiratory syndrome (SARS) outbreak and the COVID-19 pandemic underlined more negative feelings associated with worry [14,15]. Worry, which can be defined as an attempt to engage in mental problem solving or to deal with outcome uncertainty under some circumstances [16], is a central feature of anxiety disorders [17] and is associated with depressive rumination [18]. Several studies have identified worries associated with the COVID-19 pandemic, such as health-, future-, or employment-related worries and their associated consequences, such as sleep hygiene, drinking behavior, changes in social interactions, or changes in physical exercise [19,20].

For patients with a pre-existing mental health disorder or a decreased perception of well-being, lockdown measures are major stress factors affecting daily routine and social rhythms. A study by van Rheenen et al examined the mental health status of individuals with a mood disorder during the COVID-19 pandemic in an Australian sample as compared to individuals without a prior mood disorder. Their results underlined that distress in response to the COVID-19 pandemic is highlighted in individuals with a mood disorder [20]. Patients with a pre-existing mental health disorder increasingly reported worries related to infecting themselves or infecting others [21].

### COVID-19 and Psychosomatic Medical Rehabilitation

A population that has been especially concerned by the COVID-19 pandemic because of pre-existing mental health problems comprises psychosomatic patients in medical rehabilitation. They may be afraid of visiting a doctor and receiving inpatient treatment in a hospital; on the other hand, they report worsening physical and mental well-being [21]. This development aggravates the already worrisome situation of psychosocial and psychosomatic rehabilitation programs, causing patients to remain untreated. If they decide to use medical services, they are confronted with many changes in therapy programs: due to contact regulations and hygiene measures, as well as the general lack of therapists in health care systems, it is necessary to develop and establish internet-based programs and trainings as one component of therapy as well as digital support systems and platforms.

Due to the pandemic and, accordingly, its restrictions, resources had to be re-allocated and therapies had to be paused, which caused a decrease in the availability of on-site services [22]. Particularly for older people, the fear of infection can prevent hospital or rehabilitation stays [23]. Possible solutions can be home-based or telehealth rehabilitation programs [22,24], or to shift parts of the rehabilitation treatment to online preparation in the form of a home-based telehealth intervention. This is especially innovative because during the past several years, patients have frequently been prepared for rehabilitation as well as treated during the rehabilitation stay with written material. While the focus has increasingly shifted toward integrative online trainings and interventions as the basis of psychotherapy, which are considered emerging technologies in health care and therapy, so far this is rather rare in the German rehabilitation system with its focus on inpatient treatment of “chronic psychosomatic conditions at risk of resulting in long-term sickness leave and disability” (page 79 in Scheidt [25]). Such interventions and trainings are independent of time and location. They can, therefore, be used in preparation for a rehabilitation stay [26], during a rehabilitation stay to supplement and support in-person therapy [27], as well as for aftercare and stabilization processes [28,29].

Digital interventions and trainings allow for adherence to hygiene measures as well as allow for therapeutic services to be offered on a large-scale basis. In addition, patients may be offered digital treatment options if they refrain from entering a rehabilitation stay due to worries and fears associated with the current COVID-19 pandemic, such as their own health and well-being or worries associated with losing their work placement [20]. Several studies have examined the usefulness of electronically delivered cognitive behavioral therapy (eCBT), which has proven to be effective with regard to the treatment of anxiety and depression compared to regular in-person therapy [30,31]. However, it remains to be evaluated whether digital trainings and therapies are useful measures to reduce symptoms of anxiety, depression, loneliness, and perceived stress in psychosomatic rehabilitation patients and how they can be implemented in practice. A crucial aspect of digital or mobile health (mHealth) interventions is the users’ acceptance, often operationalized as perceived usefulness and ease of use. Both constructs determine the current or future usage and, thus, pose an important prerequisite for possible intervention effects [32]. Both perceived usefulness and ease of use should, therefore, be considered in mHealth interventions.

### The Health Action Process Approach

Drawing on the Health Action Process Approach (HAPA) [33,34], which is separated into motivational and volitional phases, higher intentions, planning, as well as self-regulatory strategies are needed to perform a health behavior change. During the motivational phase, an intention is formed, and after the formation, self-regulatory strategies ensure that the target behavior is realized and maintained as part of the volitional phase. Therefore, planning bridges the gap between intentions and the respective behavior [34]. Literature has shown that the HAPA as a theoretical basis for digital trainings and interventions in the sector of care after psychosomatic rehabilitation has proven to be an effective model in explaining behavior change with consequent improvements in mental health [35]. Especially for psychosomatic rehabilitation patients diagnosed with a pre-existing affective disorder, it is necessary to specifically promote competencies, such as formulating intentions, action plans, as well as coping plans, and to foster the development of outcome expectancies. Hereby, patients can increase their own control over their actions and can be supported by means of digital trainings and interventions to change from a situation-focused orientation, which is considered typical for depression, to an action-focused orientation [36]. Future research is necessary with regard to the HAPA being implemented in digital trainings and interventions for psychosomatic rehabilitation patients.

### Goal of This Study

Based on the described background, we posed a number of research questions in order to understand worries and associated consequences regarding the COVID-19 pandemic in different populations, especially medical, psychosomatic rehabilitation patients, by means of internet technology:

1. What differences are there in reported psychological variables such as depression, anxiety, loneliness, and perceived stress between the general population and patients assigned to medical, psychosomatic rehabilitation clinics? The patients were diagnosed with a mental illness and were, thus, hypothesized to be at a higher risk for an exaggeration of their illness due to the pandemic, as shown above.
2. Which worries are associated with the current COVID-19 pandemic and are there differences in the perception between the two groups? We hypothesized that individuals from the psychosomatic rehabilitation group experienced more worries with regard to the pandemic.
3. Is medical, psychosomatic rehabilitation treatment effective in terms of a decrease in symptomatology for depressive symptoms, symptoms of anxiety, loneliness, and perceived stress?
4. Do the general population and patient groups intend to make use of internet-delivered treatment components?
5. Is there a relationship between the usage, as well as perceived usefulness, of digital trainings that are offered before as well as during the rehabilitation stay in association with the intensity of mental health symptoms (eg, depression) of patients after their medical rehabilitation?

By testing these research questions, we aimed to close the research gap of evaluating mental health and COVID-19–related worries between the general population and psychosomatic rehabilitation. Furthermore, this study assessed the usefulness of internet-delivered trainings and their association with the mental health status of psychosomatic rehabilitation patients in Germany. To our knowledge, this has not been done before systematically. It is warranted to implement not only innovative but also effective internet-delivered interventions into the provision of medical services.

## Methods

### Overview

The study was conducted as part of the project “Anhand-COVID19 – Offer to achieve treatment and rehabilitation goals in compliance with hygiene and social-distancing rules” (ClinicalTrials.gov Identifier: NCT04453475), which is supported by the Dr Becker clinic group. In addition, data collection and analyses on the general population was part of the research project “TeamBaby – Safe, digitally supported communication in obstetrics and gynecology” (ClinicalTrials.gov Identifier: NCT03855735), which is funded by the German Innovation Fund (Project No. 01VSF18023) of The Federal Joint Committee (G-BA).

### First Sample: Recruitment and Procedure of the General Population

Data were collected anonymously through a nationwide recruitment campaign, press releases, social media posts, and the study home page of the TeamBaby project. No market research company or public sample was involved; however, the sample might have been selective. For data collection purposes, the software tool Unipark was used. The nationwide cross-sectional survey aimed to examine worries and coping mechanisms during the COVID-19 pandemic. All participants were informed about the purpose of the survey beforehand and provided online informed consent. Participants from the general population were not offered any form of compensation for participation. Data collection from the general population took place between May 2020 and April 2021. Time to complete the survey took, on average, 15.18 minutes (SD 11.50). Ethical approval for the online survey for the general population was given by the Ethics Committee at Jacobs University Bremen on September 17, 2019.

### Second Sample: Recruitment and Procedure of Psychosomatic Rehabilitation Patients

The second group of participants were recruited through four psychosomatic clinics from the Dr Becker clinic group and attended regular treatment at the recruiting clinics, consisting of psychological and physical interventions (ie, individual and group psychotherapy, physiotherapy, or occupational therapy) as part of the incoming process for their rehabilitation stay. The German rehabilitation system focuses not on curation but on reintegration and social participation. “Interventions in rehabilitation include psychoeducation, physical training, psychotherapy, and the training of skills particularly with regard to working ability” (page 81 in Scheidt [25]). Participants from the four psychosomatic clinics were informed about the study in writing on the hospital group's original online portal. Therefore, only patients who had access to this digital portal via smartphone, tablet, or computer before the start of rehabilitation were included. Participation was only possible after the patients had read the participation information and had given their informed consent in writing; data were pseudonymized. Rehabilitation patients were not offered any form of compensation for their participation in the online study.

**Figure 1:**
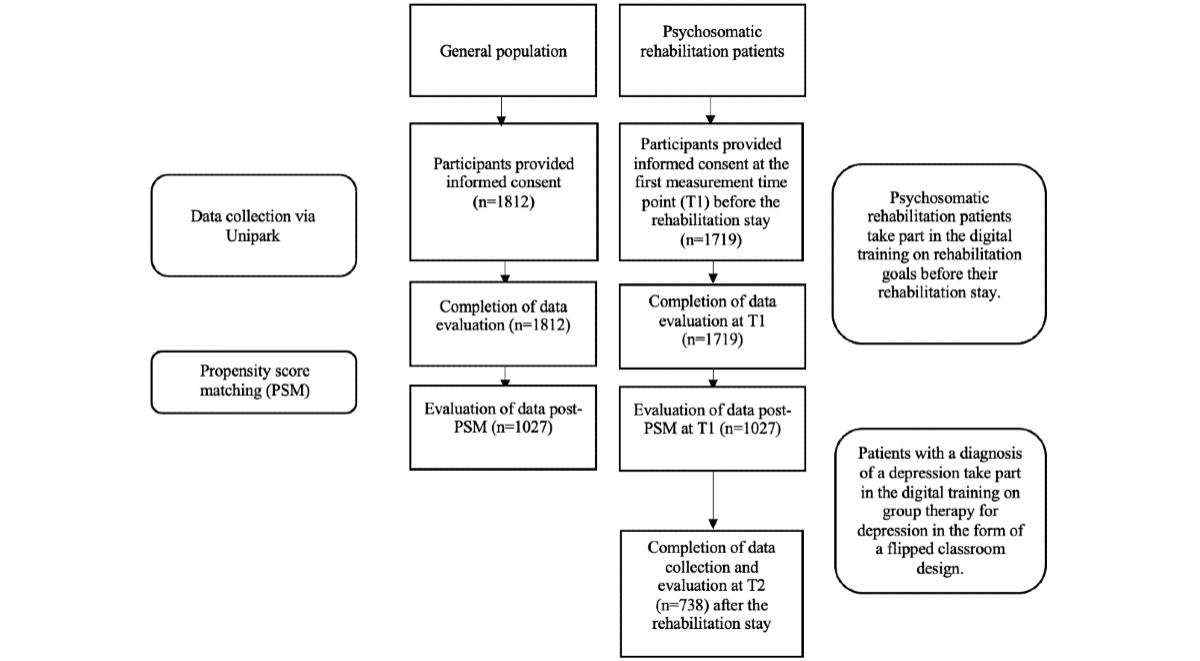
Study design of the cross-sectional and longitudiunal study.

The online survey at the psychosomatic clinics was administered between July 2020 and April 2021. Data collection at the rehabilitation clinics was longitudinal and took place at two time points: 6 weeks before the start until the first day of rehabilitation (T1) and after their rehabilitation stay (T2). Four medical, psychosomatic rehabilitation clinics took part in this study and supported the recruitment of participants as well as provided psychosomatic rehabilitation between measurement points T1 and T2. For the recruitment process and data collection process, see Figure 1. Time to complete the survey at measurement point T1 took, on average, 29.28 minutes (SD 33.10) and at measurement point T2 took, on average, 30.16 minutes (SD 52.37). Ethical approval for the online survey concerning psychosomatic rehabilitation patients was given by the Ethics Committee at Jacobs University Bremen on June 25, 2020.

### Digital Intervention Only for the Second Sample of Psychosomatic Rehabilitation Patients

Digital trainings were provided via the internet prior to patients’ rehabilitation stay to optimally prepare them for their medical rehabilitation treatment and to make good use of the treatment components, including psychoeducation, physical training, psychotherapy, and the training of skills, particularly with regard to working ability in the clinic. Such trainings could address rehabilitation goals.

The digital training on rehabilitation goals was offered to the patients in a digital PowerPoint (Microsoft) presentation training without face-to-face elements. Participants were able to participate in the digital training before their rehabilitation stay with a computer, laptop, tablet, or smartphone. The training included exercises on formulating precise plans for the rehabilitation stay. After the training, the patients were encouraged to make use of a digital exercise booklet containing exercises on formulating plans as well as for writing journal entries. Participation was on a voluntary basis. In addition, participants from two out of four psychosomatic rehabilitation clinics with a diagnosis of depression took part in group therapy for depression in the form of a flipped classroom as part of the rehabilitation treatment program (ie, digital group training for depression).

The digital group training for depression was a combination of digital and face-to-face components. The training was divided into six sessions, each consisting of a 5-minute digital training followed by a 45-minute analog group session. The digital training, including input from a therapist with flip chart accompaniment, was either viewed independently or was watched as a group at the beginning of the analog group session. Participation for patients with a diagnosed depressive disorder was mandatory. The digital group training for depression was based on cognitive behavioral therapy (CBT) and contained evidence-based components of eCBT and internet-delivered CBT interventions, based on the current state of the art [37-39]. Contents of the group sessions included, for example, an explanation of depression symptoms and how to cope with them in the form of psychoeducation, underlying models, and different available treatments (ie, pharmacotherapy and psychotherapy).

### Instruments

Table 1 provides an overview of all questionnaires and scales used for the two subsamples as part of this study.

**Table 1:** Overview of questionnaires and scales used for the general population and the psychosomatic rehabilitation patients.

Variable			General population (N=1812)	Psychosomatic rehabilitation patients (N=1719)
**Questionnaire or scale, mean (SD)^a^**				
	Overall worries related to the COVID-19 pandemic^b^		48.10 (9.07)	51.47 (7.51)
	Depression (PHQ-2^c^)		2.21 (1.89)	3.47 (1.65)
	Anxiety (GAD-2^d^)		1.95 (1.85)	3.61 (1.69)
	Perceived stress (PSS-4^e^)		7.40 (3.56)	9.44 (2.56)
	Loneliness^f^ (CES-D^g^; UCLA^h^ Loneliness Scale)		4.14 (2.12)	4.49 (1.77)
	Intention to use apps or digital trainings (HAPA^i^)		5.43 (3.24)	6.51 (3.08)
	Perceived usefulness of digital trainings (TAM^j^)		N/A^k^	5.22 (1.90)
**Sociodemographic characteristics, n (%)^a^**				
	**Age (years)**			
		≤29	407 (22.5)	70 (4.1)
		30-39	416 (23.0)	216 (12.6)
		40-49	352 (19.4)	390 (22.7)
		50-59	385 (21.2)	803 (46.7)
		≥60	252 (13.9)	236 (13.7)
	**Sex**			
		Male	529 (29.2)	602 (35.0)
		Female	1267 (70.0)	1104 (64.2)
	**Education**			
		10 or 11 years of schooling	193 (10.7)	398 (23.2)
		12 or more years of schooling	421 (23.2)	241 (14.0)
		Vocational training	507 (28.0)	791 (46.0)
		University degree	690 (38.1)	266 (15.5)

^a^Mean (SD) and frequency values before propensity score matching.

^b^Overall worries were measured by 17 items on a self-constructed questionnaire.

^c^PHQ-2: 2-item Patient Health Questionnaire; items were rated on a 4-point Likert scale from 0 (not at all) to 3 (nearly every day), with summed scores from 0 to 6.

^d^GAD-2: 2-item Generalized Anxiety Disorder scale; items were rated on a 4-point Likert scale from 0 (not at all) to 3 (nearly every day), with summed scores from 0 to 6.

^e^PSS-4: 4-item Perceived Stress Scale; items were rated on a 5-point Likert scale from 0 (never) to 4 (very often), with summed scores from 0 to 16.

^f^Loneliness items were rated on a 4-point Likert scale from 1 (not at all) to 4 (almost every day), with summed scores from 0 to 8.

^g^CES-D: Center for Epidemiologic Studies–Depression scale.

^h^UCLA: University of California, Los Angeles.

^i^HAPA: Health Action Process Approach; items were rated on a 5-point Likert scale from 1 (no, I do not intend to) to 5 (yes, and it is very easy for me), with summed scores from 3 to 15.

^j^TAM: Technology Acceptance Model; items were rated on a 5-point Likert scale from 1 (not at all useful) to 5 (completely useful), with summed scores from 2 to 10.

^k^N/A: not applicable; this item was not relevant to the general population.

#### Instruments Used for the General Population and the Psychosomatic Rehabilitation Patients

##### Worries Related to the COVID-19 Pandemic

Items assessing worries related to the COVID-19 pandemic were derived from a study that measured frequently reported burdens and worries due to the COVID-19 pandemic [40]. Consequently, an item pool of 77 elements was developed, of which 17 items were of interest for further analysis, as they described common worries related to the COVID-19 pandemic. All items were refined by psychologists and a medical professional with expertise in the field of health psychology and psychosomatic rehabilitation.

##### Depressive Symptoms and Symptoms of Anxiety

For both subsamples, symptoms of depression and anxiety were measured with the 4-item Patient Health Questionnaire (PHQ-4), which is the composite measure of the 2-item Patient Health Questionnaire (PHQ-2) [41] and the 2-item Generalized Anxiety Disorder scale (GAD-2) [42], which measure symptoms of depression and anxiety, respectively [43]. The PHQ-4 consists of four items rated on a 4-point Likert scale from 0 (not at all) to 3 (nearly every day). Summed scores of 3 or higher for both the PHQ-2 (Spearman *ρ*=0.75) and the GAD-2 (Spearman *ρ*=0.74) indicated a probable case of depression and anxiety [42,44]. The PHQ-2 and the GAD-2 were not used as diagnostic tools in this study but, rather, were used to highlight symptoms associated with depression and anxiety.

##### Perceived Stress

The Perceived Stress Scale (PSS) [45] is a globally used self-report scale that measures perceived stress; the PSS was presented to the general population and to the psychosomatic rehabilitation patients. The scale assesses “the degree to which situations in one's life are appraised as stressful” (page 387 in Cohen et al [45]), situations that are, therefore, perceived as unpredictable, uncontrollable, and overloaded during the past month. For the purpose of this study, perceived stress was assessed using the short version of this scale, the 4-item PSS (PSS-4) [46]. It assesses perceived stress by rating four items on a 5-point Likert scale from 0 (never) to 4 (very often), with a Cronbach *α* of .79.

##### Loneliness

Loneliness was assessed with two items: “How often do you feel lonely?” stemming from the Center for Epidemiologic Studies–Depression scale [47], and “How often do you feel unhappy to be alone?” from the UCLA (University of California, Los Angeles) Loneliness Scale [48] (Spearman *ρ*=0.85). The items were rated on a 4-point Likert scale from 1 (not at all) to 4 (almost every day). Both items were presented to the general population and to the psychosomatic rehabilitation patients.

##### Intention to Use Apps or Digital Trainings During the COVID-19 Pandemic

Intention to use apps or digital trainings as supportive means during the COVID-19 pandemic was assessed by rating three items on a 5-point Likert scale from 1 (no, I do not intend to) to 5 (yes, and it is very easy for me). These items were adapted based on the stages of change as part of the HAPA, which suggests that individuals typically progress through stages of behavior change independently of any time frame [49,50].

#### Instruments Used Only for Psychosomatic Rehabilitation Patients: Perceived Usefulness of Digital Trainings

Based on the different digital trainings that psychosomatic rehabilitation patients took part in, the perceived usefulness of the offered digital trainings was measured by rating two items on a 5-point Likert scale from 1 (not at all useful) to 5 (completely useful): one item for the digital training on rehabilitation goals and one item for the digital, flipped classroom, group therapy for depression. Both items were adopted and modified based on the Technology Acceptance Model, which was originally designed to evaluate patients’ responses to health information technology [32].

#### Sociodemographic and Additional Information

Additional data on sociodemographic information included participants' age, sex, and educational status. Age was categorized into five groups: ≤29 years, 30-39 years, 40-49 years, 50-59 years, and ≥60 years. Sex was categorized into three groups: male, female, and diverse. The highest obtained educational status was categorized into four groups: 10 or 11 years of schooling, 12 or more years of schooling, vocational training, and university degree. All variables were measured as categorical variables.

### Data Analysis for Both Subsamples

Literature has shown that propensity score matching (PSM) has been able to effectively reduce biases of treatment selection in nonrandomized studies [51]. Through PSM, covariates can be balanced between groups [52]. Hence, in this study, a PSM analysis was used to minimize the effect of confounding variables as well as the uneven distribution of covariates in the two groups before comparing them. The matching algorithm was based on logistic regression. Participants were matched based on sex, age, and educational status; the match tolerance was 0.01 without any failures to match.

After PSM, 2054 participants were included for further analyses, and data from the general population and the psychosomatic clinics were examined for differences. To assess whether individuals recruited from the general population and individuals from the psychosomatic clinics differed in their expression concerning psychological symptoms of depression, anxiety, loneliness, and perceived stress, a multivariate analysis of covariance was performed, controlling for gender, age, and educational level. Afterward, an exploratory factor analysis (EFA) was carried out to determine factors within the worries related to the COVID-19 pandemic based on items' factor loadings. Regarding the EFA, meaningful factors to retain for further analysis were based on the scree plot as well as the percentage of common variance explained by a given factor with an eigenvalue above 1. Meaningful factors were retained for varimax rotation. Items with a factor loading above 0.40 were used for interpretation purposes. Hence, out of 17 items primarily used to analyze worries related to the COVID-19 pandemic, one item was eliminated due to a low item loading. After EFA, significant differences between the data from the general population and the psychosomatic clinics, regarding the defined factors measuring worries related to the COVID-19 pandemic, were examined by a multivariate analysis of covariance controlling for gender, age, educational status, perceived stress, loneliness, depressive symptoms, and symptoms of anxiety.

In addition, a repeated-measures analysis of covariance was performed, controlling for gender and age on 738 psychosomatic rehabilitation patients to examine whether individuals from the psychosomatic clinics showed a change in psychological symptoms on the variables of depression, anxiety, loneliness, and perceived stress prior to and after their rehabilitation stay. To evaluate whether taking part in digital trainings (ie, rehabilitation goals and group therapy for depression) was associated with a significant change in symptom intensity with regard to depression, anxiety, loneliness, and perceived stress, a repeated-measures analysis of covariance was performed, controlling for age and gender.

To examine the intention to use common digital apps and trainings with a focus on health that were not offered during the rehabilitation stay with regard to the general population and patients from the psychosomatic rehabilitation clinics, an analysis of covariance was performed controlling for age, gender, and educational status. Finally, to evaluate the perceived usefulness of internet trainings offered during the rehabilitation stay and the association with patients' mental health status after their rehabilitation stay, a hierarchical regression analysis was performed. All data analyses were carried out using SPSS, version 27 (IBM Corp).

### Missing Data

The amount of missing data was below 5% for all items and 1.3% on average. Participants with missing data on the social-cognitive variables were included for further analysis if they had at least one nonmissing data point under the assumption of missing completely at random. However, no missing data points were imputed due to the overall low percentage of missing data points.

## Results

### Participants Before Propensity Score Matching: General Population

Overall, 3531 participants completed the online questionnaire. With regard to the general population, 1812 participants participated in the data collection. Out of these participants, 1267 (69.9%) were female and 16 (0.9%) did not respond. Age ranged from 18 to over 60 years. Out of 1812 participants, 193 (10.7%) had 10 or 11 years of schooling, 421 (23.2%) had 12 or more years of schooling, 507 (28.0%) had completed vocational training, and 690 (38.1%) had a university degree; there was 1 (0.1%) missing data point.

### Participants Before Propensity Score Matching: Psychosomatic Rehabilitation Patients

Concerning participants from the psychosomatic rehabilitation clinics, 1719 participants participated in the survey before their rehabilitation stay. Of these participants, 1104 (64.2%) were female and there were 13 (0.8%) missing data points. Age ranged from 18 to over 60 years. Out of 1719 participants, 398 (23.2%) had 10 or 11 years of schooling, 241 (14.0%) had 12 or more years of schooling, 791 (46.0%) had completed vocational training, and 266 (15.5%) had a university degree; there were 23 (1.3%) missing data points. After the rehabilitation stay, 738 participants participated in the survey.

### Participants After Propensity Score Matching: General Population

With regard to the general population of 1027 participants, 684 (66.6%) were female, their age ranged from 18 to over 60 years, 163 (15.9%) had 10 or 11 years of schooling, 173 (16.8%) had 12 or more years of schooling, 409 (39.8%) had completed vocational training, and 282 (27.5%) had a university degree.

### Participants After Propensity Score Matching: Psychosomatic Rehabilitation Patients

With regard to the 1027 participants from the psychosomatic rehabilitation clinics, 659 (64.2%) were female, their age ranged from 18 to over 60 years, 167 (16.3%) had 10 or 11 years of schooling, 194 (18.9%) had 12 or more years of schooling, 404 (39.3%) had completed vocational training, and 262 (25.5%) had a university degree.

### Difference in Psychological Symptoms

The multivariate analysis of covariance revealed significant differences in mental health between the general population and individuals from the psychosomatic clinics (*F*_4,2028_=183.74, *P*<.001, *η^2^_p_*=0.27), with age, gender, and education being significant covariates. Individuals from the psychosomatic clinics displayed significantly higher scores on all four psychological variables compared to individuals recruited from the general population: depression (*F*_1,2036_=460.51, *P*<.001, *η^2^_p_*=0.19), anxiety (*F*_1,2036_=682.11, *P*<.001, *η^2^_p_*=0.25), loneliness (*F*_1,2036_=90.31, *P*<.001, *η^2^_p_*=0.05), and perceived stress (*F*_1,2036_=424.65, *P*<.001, *η^2^_p_*=0.17) (Table 2).

**Table 2:** Descriptive statistics and mean differences^a^ between the general population and the sample from the psychosomatic clinics (ie, medical sample) across the test variables of depression, anxiety, loneliness, and perceived stress.

Test variable	General population, mean (SD)	Medical sample, mean (SD)	Mean difference	95% CI of the grand mean	*P* value	Cohen *d*^b^
Depression (PHQ-2^c^)	1.85 (1.70)	3.43 (1.64)	–1.58	2.57-2.71	<.001	0.57
Anxiety (GAD-2^d^)	1.65 (1.67)	3.58 (1.68)	–1.93	2.54-2.68	<.001	0.69
Loneliness^e^ CES-D^f^; UCLA^g^ Loneliness Scale	3.70 (1.92)	4.47 (1.75)	–0.77	4.01-4.17	<.001	0.23
Perceived stress (PSS-4^h^)	6.75 (3.42)	9.46 (2.56)	–2.71	7.98-8.23	<.001	0.30

^a^Descriptive statistics and mean differences after propensity score matching.

^b^Cohen *d*: 0.20=small effect, 0.50=medium effect, and 0.80=large effect.

^c^PHQ-2: 2-item Patient Health Questionnaire; items were rated on a 4-point Likert scale from 0 (not at all) to 3 (nearly every day), with summed scores from 0 to 6.

^d^GAD-2: 2-item Generalized Anxiety Disorder scale; items were rated on a 4-point Likert scale from 0 (not at all) to 3 (nearly every day), with summed scores from 0 to 6.

^e^Loneliness items were rated on a 4-point Likert scale from 1 (not at all) to 4 (almost every day), with summed scores from 0 to 8.

^f^CES-D: Center for Epidemiologic Studies–Depression scale.

^g^UCLA: University of California, Los Angeles.

^h^PSS-4: 4-item Perceived Stress Scale; items were rated on a 5-point Likert scale from 0 (never) to 4 (very often), with summed scores from 0 to 16.

### Analysis of Worries Related to the COVID-19 Pandemic

After varimax rotation, 16 items were retained in the analysis with factor loadings of ≥0.40 with the respective factor (Table 3). Four factors were able to explain 67.14% of the total variance. Factors identified included satisfaction with communication (six items measured on a 6-point Likert scale from 1 [completely disagree] to 6 [completely agree]; reliability indicator Cronbach *α*=.90), health-related worries (six items measured on a 5-point Likert scale from 1 [never] to 5 [always]; reliability indicator Cronbach *α*=.82), financial worries (two items measured on a 5-point Likert scale from 1 [no, completely disagree] to 5 [yes, completely agree]; reliability indicator Spearman *ρ*=0.65), and household-related worries (two items; reliability indicator Spearman *ρ*=0.28). With regard to household measures, one item was assessed on a 4-point Likert scale from 1 (not at all) to 4 (completely). The second item was assessed on a 6-point Likert scale from 1 (never or less than once per month) to 6 (daily or several times per day). Hence, the Likert scale of the second item was transformed to a 4-point Likert scale. For all factors, composite mean scores were computed.

**Table 3:** Exploratory factor analysis: factor loadings with all study participants from the general population and the medical sample (N=2054).

Scale and item	Label	Factor loadings^a^			
		1	2	3	4
**Scale 1: Satisfaction with communication**	Clear explanation	0.92	—^b^	—	—
	Early communication	0.91	—	—	—
	Sufficient information	0.89	—	—	—
	Taken seriously during communication	0.87	—	—	—
	Made sure that everything was understood	0.85	—	—	—
	Including accompanying persons and respecting situation	0.61	—	—	—
**Scale 2: Health-related worries**	Concerned about getting infected	—	0.86	—	—
	Concerned about becoming ill	—	0.84	—	—
	Concerned about visiting a doctor	—	0.71	—	—
	Concerned about infecting others	—	0.70	—	—
	Concerned about visiting the hospital	—	0.66	—	—
	Anxious when hearing news	—	0.62	—	—
**Scale 3: Financial worries**	Worries about one's job	—	—	0.87	—
	Afraid of financial difficulties	—	—	0.87	—
**Scale 4: Household-related worries**	Conflicts in household	—	—	—	0.80
	Supported each other as a household	—	—	—	0.75

^a^Exploratory factor analysis and factor loadings after propensity score matching.

^b^Factor loadings were reported for their corresponding scales.

The same factor structure was found in both samples. A total of 70.32% of the variance could be explained in the general population and 64.33% of the variance could be explained in the sample of psychosomatic rehabilitation patients.

Summarizing the results from the factor analysis, the factor structure of the evaluated worries associated with the current COVID-19 pandemic was equal across samples. Hence, the overall EFA across samples revealed four factors associated with the COVID-19 pandemic: satisfaction with communication, health-related worries, financial worries, and household-related worries.

### Differences in Worries Related to the COVID-19 Pandemic Between Groups

Results from the multivariate analysis of covariance indicated significant differences between the two groups (*F*_4,835_=17.17, *P*<.001, *η^2^_p_*=0.08) concerning worries related to the COVID-19 pandemic (Table 4): satisfaction with communication (*F*_1,838_=31.66, *P*<.001, *η^2^_p_*=0.04), household-related worries (*F*_1,838_=5.34, *P*=.02, *η^2^_p_*=0.01), and financial worries (*F*_1,837_=38.87, *P*<.001, *η^2^_p_*=0.04). Age, gender, perceived stress, loneliness, depressive symptoms, and symptoms of anxiety were significant covariates. Hence, patients recruited from the psychosomatic clinics perceived a significantly greater satisfaction with communication, increased household-related worries, but significantly lower financial worries before their rehabilitation stay (Table 4).

Patients reported being unemployed more frequently prior to their rehabilitation stay (253/1027, 24.6%) compared to the general population (123/1027, 12.0%; Table 3). Furthermore, the patient sample reported more health-related worries (Table 3). However, the difference between the groups was revealed to be nonsignificant (*F*_1,837_=0.13, *P*=.72, *η^2^_p_*=0.01).

**Table 4:** Descriptive statistics and mean differences^a^ between the general population and the sample from the psychosomatic clinics (ie, medical sample) across COVID-19–related worries.

Test variable^b^	General population, mean (SD)	Medical sample, mean (SD)	Mean difference	95% CI of the grand mean	*P* value	Cohen *d*^c^
Satisfaction with communication^d^	24.45 (7.45)	26.53 (5.69)	–2.08	25.06-25.94	<.001	0.05
Health-related worries^e^	14.56 (5.60)	15.69 (4.88)	–1.13	14.77-15.46	.02	0.04
Financial worries^f^	4.48 (2.52)	4.31 (2.21)	0.17	4.24-4.55	<.001	0.03
Household-related worries^g^	6.06 (1.32)	5.93 (1.28)	0.13	5.90-6.08	.72	0.08

^a^Descriptive statistics and mean differences after propensity score matching.

^b^Scales were aggregated from items reported in Table 2.

^c^Cohen *d*: 0.20=small effect, 0.50=medium effect, and 0.80=large effect.

^d^Satisfaction with communication: 6 items were rated on a 6-point Likert scale from 1 (completely disagree) to 6 (completely agree), with summed scores from 6 to 36.

^e^Health-related worries: 6 items were rated on a 5-point Likert scale from 1 (never) to 5 (always), with summed scores from 5 to 25.

^f^Financial worries: 2 items were rated on a 5-point Likert scale from 1 (no, completely disagree) to 5 (yes, completely agree), with summed scores from 2 to 10.

^g^Household-related worries: the first item was rated on a 4-point Likert scale from 1 (not al all) to 4 (completely) and the second item was measured on a 6-point Likert scale from 1 (never of less than once per month) to 6 (daily or several times per day). After transformation to a 4-point Likert scale, summed scores ranged from 2 to 8.

### Changes in Psychological Symptoms Before and After Rehabilitation

**Figure 2:**
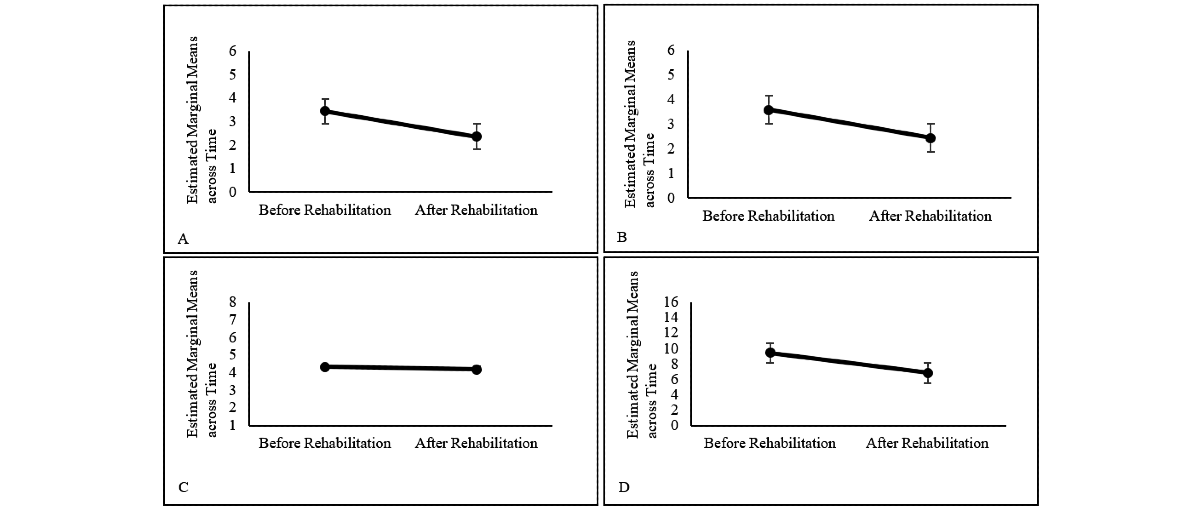
Estimated marginal means after propensity score matching for symptoms of depression (A), symptoms of anxiety (B), perceived loneliness (C), and perceived stress (D). Error bars represent standard errors of the mean.

Results of the repeated-measures analysis revealed overall significant differences across time (*F*_1,712_=23.21, *P*<.001, *η^2^_p_*=0.04). Taking a closer look at individual test variables, results revealed a significant reduction in depression (*F*_1,723_=0.98, *P*<.001, *η^2^_p_*=0.02), anxiety (*F*_1,723_=0.99, *P*<.001, *η^2^_p_*=0.01), perceived stress (*F*_1,720_=19.69, *P*<.001, *η^2^_p_*>=0.03), and loneliness (*F*_1,722_=0.99, *P*<.05, *η^2^_p_*=0.005) in psychosomatic patients after their rehabilitation (Figure 2).

### Intention to Use Common Digital Trainings and Apps

Results of the multivariate analysis of variance revealed an overall significant difference between individuals from the general population and individuals from the medical, psychosomatic rehabilitation clinics (*F*_3,2021_=51.41, *P*<.001, *η^2^_p_*=0.07). Patients appeared more inclined to use common apps and digital trainings offered outside of their rehabilitation stay supporting them in their communication with health care professionals (*F*_1,2027_=6.66, *P*=.01, *η^2^_p_*=0.01) as well as COVID-19–related health care apps (*F*_1,2027_=144.51, *P*<.001, *η^2^_p_*=0.07).

### Association Between Taking Part in Digital Trainings and Changes in Psychological Symptom Intensity

We examined whether taking part in the digital training on rehabilitation goals (ie, only digital training) and digital group therapy for depression (ie, combination of digital and face-to-face components) were associated with a decrease in symptom intensity after the rehabilitation stay compared to before. The results highlighted the following significant differences: taking part in the digital group therapy for depression was associated with a significant decrease in symptom intensity after the rehabilitation stay with regard to depression (*F*_1,725_=4.82, *P*=.03, *η^2^_p_*=0.01) and anxiety (*F*_1,725_=6.22, *P*=.01, *η^2^_p_*=0.01).

### Perceived Usefulness of Digital Trainings and Association With Mental Health Status

Table 5 shows the association between perceived usefulness of the digital trainings (ie, digital training on rehabilitation goals and digital group therapy for depression) evaluated by participants after their rehabilitation stay and their mental health status after their rehabilitation stay. Overall, increased perceived usefulness of digital training on rehabilitation goals was significantly associated with a higher reduction in perceived depression, anxiety, loneliness, and stress postrehabilitation.

**Table 5:** Association between perceived usefulness of digital trainings and mental health status of psychosomatic rehabilitation patients after their rehabilitation stay^a^.

Predictor	Dependent variable							
	Depression, *β* (95% CI)	*P* value	Anxiety, *β* (95% CI)	*P* value	Loneliness, *β* (95% CI)	*P* value	Perceived stress, *β* (95% CI)	*P* value
Participation in digital depression group therapy	.08 (–.01 to .25)	.08	.08 (–.02 to .24)	.11	.09 (–.02 to .25)	.08	.05 (–.12 to .39)	.30
Participation in digital training on rehabilitation goals	–.14 (–.37 to –.07)	<.001	–.13 (–.36 to –.05)	<.001	–.19 (–.46 to –.16)	<.001	–.19 (–.90 to –.31)	<.001

^a^Each column represents a separate analysis after propensity score matching. Analyses controlled for age, gender, and education, with gender being significant at *P*<.05 for anxiety and perceived stress, age being significant for loneliness and stress, and education being significant for education.

### Data Availability

The data that support the findings of this study are available from the corresponding author (SL) upon reasonable request.

## Discussion

### Principal Findings

In this study, differences between a sample of 1027 individuals from the general population and 1027 patients from medical, psychosomatic rehabilitation clinics in depression, anxiety, loneliness, and perceived stress during the COVID-19 pandemic were examined by means of the internet after PSM. The expression of symptoms and worries related to the COVID-19 pandemic were psychometrically assessed and tested for differences between the two samples (research questions 1 and 2). As a third research question, a potential decrease in symptom intensity on the test variables was examined for psychosomatic rehabilitation patients before starting and after their rehabilitation stay. Moreover, this paper evaluated the differences in intention to use digital apps and trainings during the COVID-19 pandemic between individuals from the general population and individuals from the psychosomatic rehabilitation clinics (research question 4). With regard to the potential decrease in symptoms, research question 5 evaluated the association between participation in digital trainings addressing rehabilitation goals and digital depression group therapy. Furthermore, the perceived usefulness of digital trainings before (ie, digital training on rehabilitation goals) and during (ie, digital group therapy for depression) the rehabilitation stay was evaluated with regard to the symptom intensity of depression, anxiety, loneliness, and perceived stress after the rehabilitation stay.

The findings from this study confirm that individuals felt affected by the COVID-19 pandemic in terms of their mental health and well-being. For individuals from the psychosomatic medical rehabilitation, symptoms of depression, anxiety, loneliness, and perceived stress were elevated compared to the general population. Thus, we can answer the research question 1 by showing that individuals assigned to medical, psychosomatic rehabilitation clinics perceive and express more mental health symptoms, which is in line with our hypothesis. In prior research, individuals with a pre-existing mental health disorder reported poorer access to support services since the beginning of the pandemic, had earlier discharges from psychiatric units, or had discontinuation of psychotherapy treatments [53-55]. The loss of such support systems due to the COVID-19 pandemic may have led to negative consequences, such as an increase in symptom intensity, increased social isolation, and perhaps even suicidal behavior [53]. Therefore, digital interventions and trainings that target positive thinking, active stress coping, and social support to reduce depression, anxiety, loneliness, and perceived stress need to be implemented for individuals with a pre-existing mental health disorder, irrespective of taking part in rehabilitation; these may also work as primary preventative measures for the general population [56]. Accordingly, research questions arise in the context of digital prevention as well as digital support interventions, which should be investigated further.

Surprisingly, psychosomatic patients perceived significantly greater satisfaction in communication with health care professionals and had significantly lower financial worries but higher household-related worries, even after statistically controlling for confounding variables. However, no significant difference between the groups was found with regard to health-related worries, which is contrary to the hypothesis that they would experience more worries (research question 2). The fact that psychosomatic rehabilitation patients perceived greater satisfaction with communication before their rehabilitation stay may be due to previous information obtained digitally (ie, through the digital training on rehabilitation goals) from the clinic as well as participating in surveys and tasks before their stay. Furthermore, contact with the rehabilitation clinics might have been perceived as an emerging support system by the rehabilitation patients, offering the hope that their situation would soon improve and that they would receive help during the pandemic. Literature has shown that effective communication with patients may prove empowering for patients [57].

The results concerning financial worries are partly in line with van Rheenen et al [20]. Their study indicated that individuals with a mood disorder expressed lower concerns with personal finances, as they were more commonly unemployed or unable to work [58]. As with the results of this study, psychosomatic rehabilitation patients increasingly indicated that they were either unemployed or unable to work before the rehabilitation stay. Due to pre-existing unemployment or lack of participation in the workforce, there was already a lower financial status and greater job insecurity as well as financial uncertainty [59]. Additionally, the inability to work due to disabilities is, in part, financially subsidized by the German social system [60]. Besides, pre-existing mental health disorders are associated with greater incapacity to work and may lead to an earlier disability pension [61]. Hence, these patients may not be aware of, nor concerned with, job uncertainty as a result of the COVID-19 pandemic due to their medical treatment, which was partially digitally supported.

Psychosomatic rehabilitation patients indicated greater worries associated with their household, which includes conflicts within the family or dissatisfaction with household dynamics before their rehabilitation stay, as compared to individuals from the general population. As the COVID-19 pandemic is characterized by strict travel restrictions, an increase in working from home and homeschooling, short-term employment, or unemployment, people tend to either spend more time with immediate family at home, leading to an increase in family conflicts, or experience isolation while quarantining. The results by Guo et al support the results of this study. They highlight that a risk factor associated with reduced mental health status during the COVID-19 pandemic is living alone [62]. Nevertheless, family conflicts as a correlate of the COVID-19 pandemic may, conversely, also be a stressor contributing to diminishing mental health. Digital solutions offer the option to bridge the gap to mobile rehabilitation, especially if family constraints prevent patients from attending rehabilitation treatment on-site. According to the research, questions arise and should be investigated further.

Moreover, individuals with and without mood disorders reported a similar frequency of worries related to health, such as worries about loved ones falling sick with COVID-19 as well as implications for one's own health and well-being. This is in line with results by van Rheenen et al highlighting equal concerns about the health and well-being of the social environment for individuals with mood disorders (ie, depression and anxiety) and those without a mental health disorder. Furthermore, individuals with and without a mental health disorder indicated almost equal concerns regarding their own health and well-being during the COVID-19 pandemic [20]. This shows that health concerns about others and oneself during the coronavirus pandemic are estimated as equally important, irrespective of the mental health status of individuals.

In addition, results indicated that for psychosomatic patients, symptoms of depression and anxiety as well as of perceived stress and loneliness decreased significantly between pre- and postrehabilitation, thus answering in favor of the third research question. This underlines the importance and necessity of medical rehabilitation treatment for patients with chronic mental disorders [63]; in particular, the group therapy for depression in the form of a flipped classroom design shows support for the decrease in symptom intensity with regard to depression and anxiety postrehabilitation compared to before the rehabilitation stay. Past evidence has shown that the combination of digital therapeutic elements and regular face-to-face therapy was able to improve the mental health outcomes of patients significantly [64-66], which is in line with the results of this study.

Interestingly, compared to individuals from the general population, psychosomatic rehabilitation patients reported a greater intention to use common apps and digital trainings focusing on health and that are not offered during rehabilitation. This offers important insights into research question 4. First of all, patients who have applied for a rehabilitation stay may already be more open to medical and lifestyle interventions. Hence, pre-existing motivation to change may foster intentions to pursue a change. Furthermore, patients with an affective disorder may be more prone to excessive reassurance-seeking, which may be defined as the repeated need for safety-related information [67,68]. Therefore, one may postulate that by reassurance-seeking through the use of health care–related apps, patients with an affective disorder may fulfill their desires for safety behaviors. However, upon the increased intention to use those apps, patients need to learn effective coping strategies and skills to perform and maintain the actual behavior without using excessive reassurance-seeking and relying on safety behaviors. Shafran et al highlighted the importance of daily self-monitoring through, for example, digital trainings in supporting patients with reduced mental health status in translating intentions into actual behavior [69].

Next to the increased intentions of psychosomatic rehabilitation patients to use digital trainings, those who evaluated the perceived usefulness of the digital training on rehabilitation goals as increasingly useful and helpful postrehabilitation stay also displayed lower symptoms regarding depression, anxiety, loneliness, and perceived stress compared to the general population. This finding clearly supports a positive association that was examined in the final research question. One might postulate that useful and helpful preparation for the rehabilitation stay provides the basis for effective digital training, such as digital group therapy for depression. Feeling well prepared, informed, and being offered additional material prior to the rehabilitation stay may motivate the patients to be an active agent and engage effectively in the subsequent digital treatment programs. Moreover, the result can be explained by the assumptions of the HAPA [33]. During the voluntary participation in the digital rehabilitation goals training, an intention to achieve a better mental health status postrehabilitation may be created. Based on this intention and in combination with the supplementary support provided during the digital training on rehabilitation goals, participants may develop adequate planning strategies to reach the desired health outcome.

With the support of the digital group therapy addressing depression, the desired behavior of achieving a better mental health status may be facilitated by means of developing coping strategies, learning new skills, and activating resources. Initial approaches in offering psychosomatic rehabilitation patients digital therapy tools for aftercare have been made by Schmädeke et al and have proven to be effective [35]. Hence, for future studies, a digital training focusing on the preparation for a medical rehabilitation stay, the support of face-to-face therapy, and empowering patients for the time after rehabilitation should be developed and evaluated based on the HAPA model, as our results are promising. In addition, such digital training should be assessed further with regard to its effectiveness in the form of a randomized controlled trial with a waiting control group.

Overall, psychosomatic rehabilitation is an effective treatment, especially during the pandemic, and should be offered to all people who either suffer from a pre-existing chronic mental disorder or who developed mental disorders due to the pandemic and its restrictions. In addition, digital trainings should be integrated with the rehabilitation process for patients with an affective mood disorder. As the COVID-19 pandemic poses several barriers toward the uptake of a psychosomatic treatment [53], it must, therefore, be ensured that people with pre-existing or newly developed mental disorders have simple, straightforward access to psychosomatic rehabilitation and additional internet-delivered supplements. Hence, possible access opportunities for psychosomatic patients may also be provided in the form of low-threshold digital trainings to offer support before a rehabilitation stay.

This study highlights the need to offer individuals support to maintain sufficient mental health, especially in times of a pandemic and its aftermath. This can be achieved in multiple, low-threshold ways that meet different needs and preferences. It may include offering individuals—not only limited to individuals with a prior mental health diagnosis to ensure prevention—facilitated access to video and telephone consultation hours, digital preventive programs, or psychosomatic rehabilitation stays. Facing a substantial lack of medical doctors, therapists, and other health care workers and the need to reduce physical contact, it is necessary to develop and establish internet-based programs and trainings as one component of therapy as well as digital support systems and platforms.

Internet-delivered treatment components offer different advantages that need to be planned more systematically. For instance, physicians or general practitioners should briefly screen all patients perceived to be at risk for stress, anxiety, or depression due to the pandemic (ie, using the GAD-2 and the PHQ-2) and then recommend further online services [70-72] or hybrid options to those with elevated symptoms. Moreover, individualized recommendations on how to deal with mental health difficulties for the general population, as well as for individuals with a pre-existing mental health disorder, should be created. Suggestions on how to deal with barriers, such as finding specialist care and waiting times during the pandemic, should be a key component of these recommendations [73], especially if individuals are confronted with hygiene regulations that might conflict with the need to connect socially with others or to seek professional help.

### Limitations and Suggestions for Future Research

Several limitations need to be considered while interpreting the findings of this study. First of all, the data evaluated from the general population are cross-sectional. Also, any changes due to the COVID-19 pandemic (ie, situation, perception, behavior, well-being, or mental health) could not have been controlled for significant events such as lockdown measures in Germany.

Furthermore, the items assessing worries related to the COVID-19 pandemic were nonvalidated items based on research found in the literature so far. Hence, for future studies, the analyzed items should be examined with respect to validity, while this study provides the first results regarding reliability. Another critical point to highlight is that mental health, in the form of symptoms of depression, anxiety, loneliness, and stress, was self-reported. In addition, the questionnaires that were used (ie, the PHQ-2 and the GAD-2) only gave an indication about the symptom intensity but were not used as diagnostic tools.

Furthermore, we had no indication of symptoms or clinical mental health diagnoses before the start of the COVID-19 pandemic. Hence, we cannot be certain that the levels of psychological symptoms reported by participants were subject to the COVID-19 pandemic. However, it has been suggested that symptoms of anxiety and depression increased as a result of the COVID-19 pandemic compared to historical normative data [74].

In addition, the digital training on rehabilitation goals and group therapy for depression were not tested with regard to their effectiveness beforehand. Furthermore, with regard to the data collected with the psychosomatic clinics, it remains to be evaluated in future research whether somatic aspects, including a potential COVID-19 infection, are a confounding factor for the expression of symptoms of depression, anxiety, loneliness, and perceived stress.

### Conclusions

This study provides insights into the mental health status and perceived well-being of psychosomatic rehabilitation patients compared to the general population during the COVID-19 pandemic. In addition, this study was able to evaluate COVID-19–related worries between the general population and psychosomatic rehabilitation patients. Further, the usefulness of internet-delivered trainings and their association with the mental health status of psychosomatic rehabilitation patients in Germany was assessed. The results suggest that psychosomatic rehabilitation patients perceived greater symptoms of depression, anxiety, loneliness, and stress compared to the general population before their rehabilitation stay, which validates their status as assigned to rehabilitation. Future studies should replicate these findings in other countries and with individuals from a different cultural background. In particular, the question remains as to whether different health care systems and rehabilitation treatments (eg, delivered at a higher proportion in a mobile, internet-delivered mode) would result in the same outcomes. It is also imperative to disentangle which components of the internet-delivered interventions were especially effective in which patients.

The general population perceived greater financial worries, whereas patients before their rehabilitation stay perceived greater worries associated with their health and household. In addition, our results underline that in comparison to before their medical rehabilitation stay, patients’ symptoms of depression, anxiety, and perceived stress were significantly lower after their rehabilitation stay. This stresses the value and necessity of psychosomatic rehabilitation treatments, concerning the psychotherapy of chronic mood disorder, and their relevance during the COVID-19 pandemic, especially among individuals with elevated symptoms and needs.

Internet-delivered medical rehabilitation components integrated into face-to-face therapy have the option to accelerate mental health improvements due to rehabilitation, which is especially important in times of limited treatment capacities and the need to reduce the transmission of viruses (ie, physical contact between treatment providers and patients). Internet-delivered medical treatment can bridge the gap and can also help patients to cope with a potential aftermath of the COVID-19 pandemic in terms of more patients in need of care than available resources at the patients’ residence.

## Abbreviations

CBT: cognitive behavioral therapy
eCBT: electronically delivered cognitive behavioral therapy
EFA: exploratory factor analysis
GAD-2: 2-item Generalized Anxiety Disorder scale
HAPA: Health Action Process Approach
mHealth: mobile health
PHQ-2: 2-item Patient Health Questionnaire
PHQ-4: 4-item Patient Health Questionnaire
PSM: propensity score matching
PSS: Perceived Stress Scale
PSS-4: 4-item Perceived Stress Scale
SARS: severe acute respiratory syndrome
T1: first time point (6 weeks before the start until the first day of rehabilitation)
T2: second time point (after rehabilitation)
UCLA: University of California, Los Angeles
WHO: World Health Organization
